# Physiology

**Published:** 1826-04

**Authors:** 


### Quarterly Periscope

OF

### PRACTICAL MEDICINE;

BEING

### The Spirit of the Medical Journals,

*Foreign and Domestic;*

WITH COMMENTARIES.

### I.

### Physiology.

——— latas à numine leges
Religiosa doce, mentesque cupidine veri
Allice.

1. *M. Pouillet on Atmospheric Electricity.* Various theories have been formed by meteorologists, to account for the electricity sensibly present in the atmosphere. Of these, Volta's was, perhaps, the only plausible one. That philosopher was induced to believe, that bodies, in passing from one state to another, undergo a change in their electric condition; and he supposed that the electricity lost in storms, was continually being renewed by that produced by evaporation perpetually going on from the surface, as well of the land as of the water.

The recent and interesting researches of Pouillet were instituted, not merely to ascertain the truth of the Italian professor's hypothesis. he was also desirous of discovering the efficiency of another cause, which he believed to be of no small importance in the production of electricity, and of bringing to proof a theory of his own, relative to the distribution and accumulation of this principle in the atmosphere.

Numerous and various experiments have brought him to the conclusion, that the mere passage of a body from the solid form to a state of vapour, is unaccompanied by the development of electricity—that the result is similar when vapour is condensed either into the liquid or solid form.

He conceived that Volta, though too accurate an observer to be mistaken as to the fact of the presence of electricity in his experiments, was nevertheless, deceived as to the cause of its production, by the formation of carbonic acid, which mixed with the vapour of water and complicated his experiments.

In 1782, Volta, Lavoisier, and Laplace shewed, that electricity was developed during chemical action, but as experiments relating to this point, are liable to afford different and contradictory results, from slight differences of circumstances, the question has been regarded as rather undecided.

It became, on this account, an object of especial attention with M. Pouillet: he finds that, in the combustion of charcoal, there is an unequivocal production of electricity—that the acid produced is in the positive state, whilst the charcaol always becomes negative. It is necessary, in order uniformly to obtain the same results, that the combustion should take place only at the upper part of the piece of charcoal, and by no means extend over the whole of it; otherwise the contact both of the charcoal and of the carbonic acid with the plate of metal, destined to receive the electricity, will render the experiment irregular. To discover whether the electricity rendered evident in the preceding experiment, was to be attributed to chemical action, or to the conversion of the charcoal from the solid to the gaseous state, he examined the flame produced by the combustion of hydrogen.

The external part of the flame constantly exhibits positive, and the interior negative, electricity:—a transfer of electricity taking place between the molecules which are combining, and those which are about to do so.

This fact is supported by a great number of experiments on the combustion of phosphorus, sulphur, the metals, alcohol, æther, fat substances, and vegetable matter.

As plants, during vegetation, exert a chemical action on the atmosphere, sometimes converting its oxygen into carbonic acid, and at others, decomposing the carbonic acid already existing in it, the idea suggested itself, that if electricity were developed in these processes of vegetation, their very extensive operation would warrant one in attributing to them, a considerable portion of the electricity of the atmosphere. To investigate this subject, M. Pouillet examined the vegetation of seeds, in an insulated situation, having a condenser connected with the soil. Till the germs appeared at the surface, no signs of electricity could be detected, but as vegetation advanced, it became very evident. For the success of this experiment, it is necessary that the air should be in a state of considerable dryness. When this does not happen to be the case, the apartment must be artificially dried by quick-lime, or some other absorbent. It is obvious, that the soil could not acquire one electric state, without the opposite state, in a corresponding degree, being communicated to the atmosphere. If, then, a languid vegetation on a surface of five or six square feet, be capable of producing very decided effects, may we not reasonably conclude, that the influence of the same cause, operating over a large portion of the surface of the earth, is fully adequate to the production of many of the phenomena which we observe?

A second memoir of the same author carries the subject still farther, and exhibits other causes, besides the process of vegetation which contribute to supply the atmosphere with electricity.

In the first memoir, he had shewn, that when two bodies combine, electricity is developed; in the second, he proves, that similar phenomena attend the separation of bodies which were previously combined, and he applies this fact to the numerous instances of decomposition, which Nature is spontaneously producing on the surface of our terraqueous globe.

M. Pouillet, in his experiments connected with this enquiry, employed two processes. The first resembles that adopted by Saussure, in his experiments on evaporation, and consists in connecting one of the disks of the condenser with the heated vessel in which the subject of the experiment is to be placed. By the other process, the heated vessel is dispensed with, and he makes use of one of Frefnel's large lenses to heat the body, whilst it rests on a plate of platina.

It should be remarked, that when vessels of copper or iron, or of other materials, on which the substances under examination can act chemically, are employed, the result will be a complication of effect by which the phenomena will sometimes be heightened, and at others neutralized.

The results of his experiments, are 1st, That by mere evaporation, as before stated, whether it be rapid or slow, no signs of electricity are produced. 2nd. That evaporation from an alkaline solution, however weak, whether it be of soda, potash, barytes, or strontian, leaves the alkali electrified positively. 3rd, That when other solutions, whether saline or acid, are employed, evaporation leaves the body which was combined with the water electrified negatively.

Of the numerous saline solutions which were essayed, that of muriate of soda, was naturally the one which excited the greatest interest. It formed no exception to the rule—hence it can hardly be doubted, that evaporation from the surface of the sea, forms one of the most important sources of atmospheric electricity. Even lakes and rivers must have their influence, since their waters are never perfectly pure.

The preceding researches are not less interesting to the physiologist and to the physician, than to the meteorologist. Medical meteorology is a subject which has hitherto obtained far less attention than it deserves. Observations on the barometer and thermometer alone are by no means adequate to explain the varied influence which the atmosphere exerts upon the animal œconomy. The hygrometric condition of the medium in which we are placed, and its states of motion and rest, are manifestly productive of most important results, and it can hardly be doubted, that these are materially modified by the electric state of the atmosphere, since every day seems to add strength to the opinion, that electricity is not merely a powerful excitant of vital action, but is essential to many of its phenomena.

We think it by no means improbable, that accurate meteorological observations, embracing the different points to which we have alluded, might trace much of the medical effect of sea air to the principle which M. Pouillet has developed. We have ourselves experienced the striking difference of effect between the Sirocco as a land, and as a sea wind, a difference which is doubtless to be attributed to a change in its hygrometric and electric states.

2. *Biliary Secretion.* Much disquisition has taken place respecting the question, whether the bile is secreted from the vena portæ, or from the blood conveyed by that vessel, and the hepatic artery in common. The more general opinion was that the portal blood furnished the bile, while the hepatic artery acted as the nutrient vessel of the liver. Yet this doctrine was doubted, especially as Mr. Abernethy had found in the body of a child, apparently well nourished, the portal vein terminating in the inferior cava, without passing through the liver. Such anomalies as these, however, are very bad bases for physiological deductions, since Nature often compensates for the loss of function in one part, by additional function in another.

We do not recollect such decisive experiments to ascertain the point in question, as those lately instituted by Dr. Simon (*Nouv. Bullet. de la Societ. Philomath. Aout*, 1825) in which ligatures were put on the vena portæ, hepatic artery, and biliary ducts of animals.

1$^{mo}$. When the excretory ducts alone (of the pigeon) were tied, the liver became gorged with bile, and deeply tinged. In from ten to twenty hours the animal passed, per anum, a quantity of fluid resembling that by which the liver was gorged. This fluid was found to exist only in the cloaca, and was, therefore, thrown out by the exhalent arteries of the part.

2$^{ndo}$. When the hepatic artery and the ducts were tied, the liver took on the same deep tint—and bile was found in the cloaca as before. Hence it was proved that the liver secreted bile, long after it ceased to receive blood from the hepatic artery.

3$^{tio}$. The vena portæ was secured, as was also the ductus communis. The liver remained colourless—no trace of bile was found in it, the ducts, or the intestine. The cloaca was filled with excrements, shewing no admixture of the colouring matters seen in the former experiments. These trials were repeated several times, and with the same results.

---

3. *Report to the Academy of Sciences, on Dr. Barry's Memoir on the Circulation of the Blood in the Veins.* By M. DUMERIL.

M. Dumeril first alludes to the great variety of opinions which have divided the physiological world on this subject for many years. Previous to Bichat, however, it was a pretty general opinion, that the cause of the motion of the blood in the veins was to be sought in the *vis à tergo* from the heart. This celebrated physiologist, on the other hand, maintained that the venous movement was the result of the absorbent faculty of the venous radicles. Some, since Bichat's time, have endeavoured to explain the venous current by the pressure of the muscles and other organs on the vessels themselves; but many have looked upon the circulation in the venous system as intimately connected with the function of respiration. Haller, in particular, had remarked that, during a strong inspiration, the veins became pale and void of blood, while, at the moment of expiration, they swelled and became of a darker colour. Morgagni made a number of experiments, with the view of ascertaining the relation between respiration and venous circulation; and afterwards, Magendie proved the fact of acceleration of the current during inspiration, but did not attempt to account for the phenomenon. Bichat himself, was far from being satisfied with his own account of the mechanism of the return of blood from the arteries, and acknowledged that there was something mysterious in the matter. Dr. Barry, in his memoir on this subject, proposes, 1mo. To determine by positive experiments what is the cause of the blood's motion along the veins towards the heart.—2ndo. To ascertain the relative velocity of the sanguineous current in the veins and in the arteries.—3tio. To prove that the supposed constant stream in the veins cannot be accounted for by the causes hitherto attributed to that phenomenon.

As to the first proposition, M. Dumeril remarks, that Dr. Barry has exhibited conclusive evidence, by means of the most ingenious and novel experiments, that the blood moves along the veins *only* during inspiration (que le sang ne traverse jamais les veines que dans le temps de l'inspiration)—shewing, at the same time, that all the phenomena of the venous circulation, both in man and in animals of similar structure, can be explained by the mechanical action of atmospheric pressure. In respect to the second point, (the assertion of the experimenter, that the frequency of the pulse does not afford direct proof of the rapidity of the circulation, but merely indicates the manner in which inspiration succeeds to exspiration)—the position is not proved, says M. Dumeril, by unobjectionable experiments, but rests only on reasoning. Finally, in regard to the third point, the commission has to remark, that Zugenbuhler, in the year 1815, asserted, that the circulation in the veins was owing to atmospheric pressure, in consequence of *active dilatation* of the chambers of the heart—whereas, Dr. Barry attributes the dilatation of the heart to the attempt at a vacuum which is in operation throughout the whole thoracic cavity. The latter (Barry) proves his position by direct experiments—the former (Zugenbuhler) supports his doctrine only by arguments. The report concludes with a resolution, that Dr. Barry's memoir shall find an honorable place in the Archives of the Academy—that it is full of merit—and that the author ought to prosecute his experiments on the absorption of poisons, and on the means of preventing the absorption of deleterious substances applied to the surface or to wounds—researches which are corollaries of his doctrine of the venous circulation.—*Bulletin des Sciences.*

Till we are favoured with the whole of the memoir, and can examine the experiments on which the doctrine is founded, we shall abstain from all remarks. We are not without hopes, however, that the present investigation will throw some more light on the circulation of the blood—a phenomenon which is by no means explained by any experiments or hypotheses which have hitherto been executed or proposed by physiologists, however easy and clear it may seem to those who take for granted what their masters in the schools have taught them.

4. *Spectral Illusions.* We were much amused with a case of this kind, related by Dr. Crowther, in a cotemporary Journal. The patient was a rough north-country lass, who, on coming to reside at Wakefield, where she had little to do and much to eat, became as fanciful as any of her betters in the highest walks of life. Hysteria was the first physical effect—then tetanus, (if tetanus it could be called) and lastly, a vision from the realms of the dead, in the shape of her master's late wife, who delivered her a message to her master. Here Dr. Crowther enters into a very profound psychological discussion, whether or not ghosts can talk. He is greatly disposed to think they cannot hold mortal parlance; and considers this negation proved by—" the common experiment of a bell, *in vacuo*, which is incapable of emitting sound." By assuming, says he, that the departed immaterial spirit could give no impulse to the air, and, therefore, produce no sound—" so far the effect produced by the immaterial principle would resemble the bell, *in vacuo*." " If, therefore, a departed spirit cannot effect atmospheric vibration, it is impossible for it to perform the more difficult operation of talking, without lungs, without wind-pipe, without tongue, and without nerves."—*Ed. Journal, p.* 53. Really we wonder, that a man of Dr. Crowther's sense, should enter into such a ridiculous question, as that of the possibility of departed spirits holding conversation with living beings—and especially with ignorant servant maids.

---

5. *State of the Saliva in Mercurialization.*. Dr. Bostock, in the last volume of the Medico-chirurgical Transactions, has stated some experiments on the above subject. Some years ago he analyzed the saliva, and found two animal substances in this fluid—one of them similar to albumen in its coagulated state—the other resembling the uncoagulable matter of the serosity of the blood. The *first* is in union with, but not soluble in water—it is not coagulable by heat, nor precipitated by the various chemical re-agents which act upon albumen, but affected by nitric acid and potash, in the same manner as albumen. The other animal ingredient in saliva is characterized by its not being acted upon by the various substances which coagulate or precipitate albumen, while it is precipitated by sub-acetate of lead, and by certain salts of tin and silver. Such being the state of natural saliva, Dr. B. embraced an opportunity of examining this fluid while the system was under the influence of mercury, and while about two quarts *per diem* were discharged.

We shall not recapitulate all the qualities or appearances observable in this saliva, which seem to be needlessly minute. It was soluble in water—did not indicate acid or alkali—quantity of solid contents, on evaporation, about one-fiftieth of the whole weight. Exposed to a boiling heat, a degree of coagulation was produced, but there was no precipitate of solid matter. It was submitted to the following tests :—" Solution of corrosive muriate of mercury produced a considerable precipitate, and, when the mixture was subjected to the heat of boiling water, a number of dense flakes separated from it, leaving the fluid transparent; after being passed through a filter, it had the aspect of pure water. By the addition of muriatic acid, the opacity of the saliva was considerably increased, and by applying heat, a coagulum was formed which gradually subsided, but it was less firm, and the separation was less complete than when corrosive muriate of mercury had been employed." From these experiments we learn that the chemical constitution of the mercurial, is different from that of common saliva—the difference consisting chiefly in its containing a quantity of animal matter, possessing properties similar to those of albumen in its uncoagulated state—or, as it exists in the serum of the blood. Further experiments shewed that there was no appreciable trace of mercury in the saliva—hence it follows (as our author reasonably concludes) that the effect of this medicine on the salivary glands must be through the system generally—and hence, too, we may conclude that all the organs destined for the secretion of mucus, undergo the same change. "This change would appear to consist essentially in the conversion of the animal matter, from the state of a mucous to that of a serous, or rather of an albuminous fluid."—The following speculations we give in the words of the author.

"As we find that at least one operation of mercury is to convert a mucous into a serous secretion, the following queries suggest themselves; whether we may not conceive that the action of this remedy, in the cure of glandular obstructions, consists simply in producing this change in the nature of the secretion? Whether, even in the removal of the diseases of surfaces, mercury may not operate upon the same principle, by counteracting the effect of specific secretions, and reducing them to the mere transudation of a serous fluid? Whether by examining the effects of the remedy upon the chemical nature of the mucous secretions, we may not be furnished with a more accurate test of its constitutional action, or at least of the extent of this action, than we at present possess in the mere quantity of saliva that is discharged?" 81.

Whatever may be the chemical effects of mercurial action on the system, the physiological are very remarkable, and far from being generally understood. The most prominent are (we mean when ptyalism takes place) great irritability of the system, partly from the soreness of the mouth—considerable absorption of all parts of the body, both sound and morbid—a corresponding extenuation of the body—increased discharge from all the secretory organs, including, we believe, the skin—an almost invariable change in the biliary and alvine secretions from a morbid (if previously morbid) to a healthy condition—an increased proportion of yellow bile in the motions, if before healthy—a relish for food, but great misery in taking it, if the mouth be very sore. All these effects go on, with greater or less activity in different constitutions, as long as the ptyalism lasts. When this is over, a new order of things takes place. The appetite becomes almost ravenous—every kind of food is devoured with a zest quite extraordinary, and fully equal to that which we have all felt in our school-boy days, when the digestive organs were in prime operative function. Absorption and secretion now slacken their pace even below par; while nutrition and deposition increase in proportion, and go on till the body is built up considerably beyond the point at which it stood previously to the exhibition of mercury. The functions of assimilation, absorption, and secretion, then come to an equilibrium, and the healthy standard remains till some new source of derangement occurs. This portrait is not drawn from individual feelings and experience alone, but from a very wide range of experience in the effects of mercury exhibited for syphilitic, hepatic, dysenteric, and other affections—as well as where ptyalism has been brought on by accident, and undesignedly. Strange as it may seem to many practitioners of the present day, we have rarely or never seen any bad effects resulting from mercury, carried to moderate, or even pretty severe ptyalism, in the above manner. The injurious effects which we have witnessed were, sometimes ulceration of the tongue, and exfoliations of the alveolar process, when the ptyalism ran to an outrageous extent—but much more frequently we have seen the bad effects of minute doses of mercurials taken for weeks and months in succession, and where the mouth was never made sore. It is in this long-continued exhibition that the stomach becomes dyspeptic—scrofula developed—and the nervous system injured. When mercury is given, it should only be for a few days, or, at the most, weeks—and that, in doses sufficient to act on the abdominal secretions pretty actively. It should then be omitted, at least, for a time. When it is desirable to affect the system constitutionally, the patient should be kept strictly confined, and the constitutional effect obtained, if possible, in the course of a few days, or of a week. It is then to be maintained so long only as may be deemed proper for the removal of whatever complaint it was exhibited for. These observations are few and brief, but they are drawn faithfully from nature, and may not be undeserving the consideration of the practitioner.

